# Gamma-glutamyltransferase, possible novel biomarker in colon diverticulosis: a case-control study

**DOI:** 10.1080/14756366.2018.1428802

**Published:** 2018-01-26

**Authors:** Constantin Bălăeţ, Bogdan Ioan Coculescu, Gheorghe Manole, Maria Bălăeţ, Gabi Valeriu Dincă

**Affiliations:** a Faculty of General Nursing, Bioterra University, Bucharest, Romania;; b Lil Med Clinic, Bucharest, Romania;; c Faculty of Medecine, Titu Maiorescu University, Bucharest, Romania;; d Center for Military Medical Scientific Research, Bucharest, Romania;; e Colentina Clinical Hospital, Bucharest, Romania;; f Imperial College London, London, UK

**Keywords:** Gamma-glutamyltransferase, inflammation marker, colon polyposis, oxidative stress

## Abstract

The gamma-glutamyltransferase (GGT) is recognized in medical practice as a useful indicator for the detection of liver lesions, especially those induced by the excessive consumption of alcoholic or cholesterol-associated drinks. The present study, although it includes a very small number of cases diagnosed with colon diverticulosis-diverticulitis associated with polyposis at the same intestinal level, identifies the presence of increased circulating concentrations of this enzyme in the serum. Its serum levels are tracked “dynamically” throughout a year after the diagnosis and start of the therapy. The study calls into question the release of the enzyme from the edge of the enterocytes’ brush-like edge, leading to the pathogenic disturbance of regional redox homeostasis. The hypothesis gives the circulating values of GGT predictive value for cellular oxidative stress, as well as for indirectly expressing the glutathione level in cytosol.

## Introduction

The γ-glutamyltransferase (GGT or γ-GT) is a glycoprotein-enzyme predominantly linked to cell membranes in tissues and organs that possess a high secretion or absorption capacity^1^. This assumes that beyond its predominantly hepatocytic localization is also present in the intestine, kidneys, pancreas, myocardium, etc. Regardless of the nature of these cells, it is widely accepted that GGT levels may increase as a result of membrane instability or apoptosis destruction^2^^,^^3^.

The physiological development of digestive-absorption processes in the enterocytes is dependent, amongst other factors, on the tissue concentration of glutathione^4^. This is because GSH is part of the protein complex that controls the activity of reactive oxygen species (ROS) through GSH/GSSG (GSH = reduced form of glutathione; GSSG = glutathione disulfide or oxidized glutathione). Maintaining the glutathione homeostasis at the enterocyte level is the attribute of G-GTP-ase, a heterodimer enzyme, which participates in the γ-glutamyl cycle. Within the cycle, the reduced glutathione antioxidant tripeptide (GTH) is transported to the extracellular membrane (where the active enzyme centre is located) to be cleaved into its compounds: cysteinyl-glycine and γ-glutamyl residues. Subsequently, these radicals can be transferred to other amine acids or peptides^5^^,^^6^. Disturbance of redox homeostasis at the enterocytic level leads to malfunction of the γ-GT enzyme activity at microvilli.

## Material and methods

Between 2015 and 2016, out of the 1600 patients treated for various conditions through the gastroenterology department at Lil Med Clinic, we retained for study 26, who were diagnosed with: solitary colon polyps (1 or 2) and diverticulitis, as a complication. The cases presented diverticulitis in its uncomplicated form: three cases were acute, and the rest chronic, explaining the nonspecific symptomatology reported by patients. A total of 16 of these were males and 10 women. Patients were aged 55–65 years.

Knowing that serum levels of GGT are increasing in various circumstances and conditions, a constant concern was to provide a batch for study which is homogeneous. Thus, the patients who smoked were not admitted to the lot, and only occasional alcohol consumers. For the reliability of the blood-dosing result of GGT, all patients were banned from consuming alcoholic beverages for at least 10 days, knowing that the enzyme had a 3–5 day lifetime.

Based on anamnesis, but also on the laboratory tests, it was established that none of the patients had diabetes, chronic obstructive pulmonary disease, inflammatory bone disease, thyroid functional disorder, or chronic hepatopathy, regardless of its clinical form, including the presence of mechanical or functional cholestasis. In the past two months, no patient in the study followed therapy with hypolipidemics or with other drugs such as barbiturates, steroids, etc., which would cause an increase in serum levels of GGT, amongst other adverse reactions.

Based on reports from the literature that serum levels of GGT are closely associated with a high mortality rate due to ischemic cardiopathy, all patients underwent resting ECG and Master Exercise Test. This, together with the dosage of serum levels of troponin and serum levels of some cardiomiochemicaly released isozymes (CK-MB, LDH_1_) allowed us to rule out the possibility of myocardial ischemia, which would have contributed to the increase in serum concentration of GGT^3^^,^^7^^,^^8^.

The diagnosis was established following the clinical examination and based on the results of laboratory and paraclinical investigations. Paraclinically, the 26 patients enrolled in the study underwent: abdominal ultrasound, conventional colonoscopy, and abdominal CT. The final diagnosis was based on the findings of the colonoscopic examination and the result of the histopathological examination of the biopsy taken. After resecting the polyps, the cases were monitored for one year, performing:

- quarterly control: blood biochemical and haematological laboratory tests;
- after one year, repeat colonoscopy.
- Based on the results of laboratory tests on blood biochemistry, five common features were present, in all 26 patients in the batch:
- existence of erythrocyte sedimentation rate (ESR) values (determined at 1 h) above the normal upper limit;
- the presence of a mild anaemia, which morphologically embraces the appearance of microcitic anaemia, hypochromic; aetiology confirmed by the positivity of occult haemorrhage tests in faeces.
- positive test for quantitative dosing of reactive C protein, in the context of which tests for dyslipidaemia revealed normal or insignificant increases of up to 10% above normal (borderline);
- circulating concentrations higher than normal for γ-GT;
- the circulating level of fibrinogen and α_2_-globulins admitted in the literature as markers of inflammation, just like that of reactive C protein;
- positive Adler test: the test was to be performed as a screening probe to identify the occurrence of occult bleeding in the faeces, in compliance with all diet standards in the days preceding the analysis.

These lab investigations were considered “target” tests, determining them quarterly for one year.

## Results

At the time of establishing the positive diagnosis of the associated colonic conditions, the mean values of the “target” laboratory tests were:

- haemogram: normal parameters except haemoglobin (Hb) with oscillatory values between 10.2 and 11.7 g% ml); the calculation of erythrocyte constriction values allowed to diagnose the type of anaemia as microcytic, and hypocromic;
- ESR: with values between 25 and 39 mm/1 h; except for three cases at 43 and 50 mm/1 h, respectively;
- reactive C protein: >8 mg% ml;
- serum γ-glutamyl transpeptidase: up to 300 U/l;
- the occult haemorrhage test was positive in 85% of patients.

**Figure 1.**
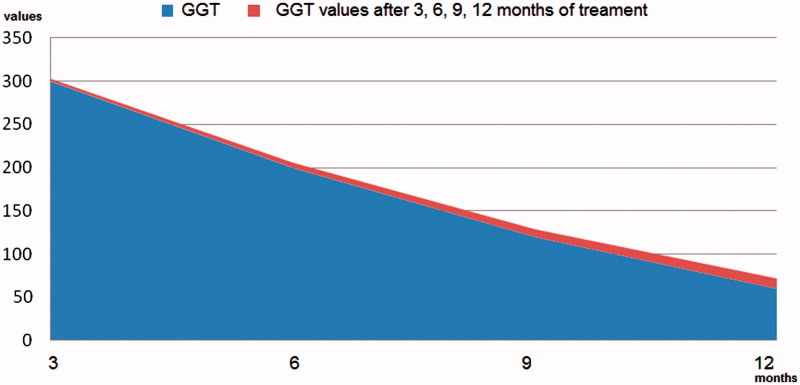
Diagram of serum concentration values of GGT, determined during the four monitoring trimesters of patients.

The objective of the study was to track the evolution of the serum values of these “target” tests, but especially of GGT, in one year. For this purpose, we admitted that, depending on the dynamic evolution of the circulating level of GGT and its corroboration with the results of the other laboratory tests, as well as the one-year colonoscopic appearance, it can be accepted as a possible marker of inflammation, inflammation that most likely affected the integrity of the enterocytic membrane. This was because the incrimination of colon polyps could not explain the evolution of serum values of GGT, which during the year that followed the endoscopic resection decreased progressively but slowly (Figure 1).

## Discussions

In terms of clinical examination, the first consultation with the gastroenterology cabinet was requested for: neurastheniform condition, dyspeptic phenomena associated with the presence of a mild anemia, apparently without cause. The particularity of the clinical picture required, among other things, the performance of a colonoscopy, beyond the other haematological–biochemical laboratory investigations included in the exploration protocol.

In patients in the study, monitoring of the target test values allows us to support:

- that there was a normalization of circulating Hb (without martial therapies), of ESR and of reactive C protein at different times, ranging from 6 to 12 months after the removal of the polyp. We mention that we have not instituted martial therapy, because relatively recent experimental studies indicate that GGT can stimulate the synthesis of oxidative species in contact with iron ions or other transition metals, which would maintain its increasing circulating concentration^9^.
- the existence of the GGT return trend towards the maximum permissible normal circulating limit (men <61 U/l; women <36 U/l) but at the end-of-year no patient had normal serum values (Figure 1).

Also, a positive correlation was found between serum concentrations of GGT and reactive C protein, suggesting the persistence of diverticular inflammation, association that can be compared to that reported by the literature and admitted as a marker of atherogenesis, a condition for which cross-compliance with risk metabolic factors was accepted^10^. There was no possibility of correlating the circulating levels of the enzyme with the concentrations of fibrinogenemia and serum α_2_-globulins.

In medical practice, it is notorious that GGT is the enzyme with the highest sensitivity in detecting liver cholestasis, regardless of its aetiology (mechanical or functional) and the presence or absence of cholangitis or cholecystitis^5^^,^^11^. Numerous other epidemiological observations support the relationship between serum activity of GGT and mortality through cardiovascular events, diabetes mellitus, or various metastatic neoplasias^4^^,^^8^^,^^11–13^.

Although, almost unanimously, it is recognized that GGT that is dosed in the serum is derived from the liver, which is linked in particular to HDL and LDL, the biochemical laboratory can dose all four fractions under which the enzyme is found: b- γ-GT, m- γ-GT, s- γ-GT, and f- γ-GT^1^^,^^14^.

In our study, we interpret the serum increases of GGT as coming from the enterocyte membrane level through three possible mechanisms: from the diverticular inflammatory process, from membrane lesions developed by the action of detergent exerted by bile salts that did not undergo physiological metabolism up to colon and apoptosis of enterocytes and cells migrated to the diverticular wall, but also to the proliferated polyp generators. Petrogenetically, the main source contributing to increased serum enzyme levels is the presence of diverticulitis. We support this on the basis of the evolution of serum values of GGT which, although progressively reduced, did not normalize even if polypectomy was practiced. Failure to achieve the normal GGT normal values even after one year, even if the patients underwent treatment for diverticulitis, means that the inflammatory process is not yet therapeutically controlled, which allows it to contribute to the release of the enzyme. Admitting this source, we argue that the assay for serum concentration of GGT gives the enzyme inflammatory biomarker value: circulating high levels indicating the presence of inflammation.

Based on the data from the studied medical literature, the role of the enzyme involved in the diverticulitis inflammatory process is explained by pathogenic intervention of the reactive oxygen species generated regionally, which activates a sequence of reactions. In physiological concentrations, the cellular level interferes with oxide-reduction reactions, *via* glutathione, allowing for the normal functioning of various cellular components, including membrane lipids, including arachidonic acid. Installing a glutathione deficiency is also a source of regional oxidative stress, a promoter of inflammation development. The result is damage to the membrane-lumen surface integrity of enterocytes that is rich in GGT.

**Figure 2.**
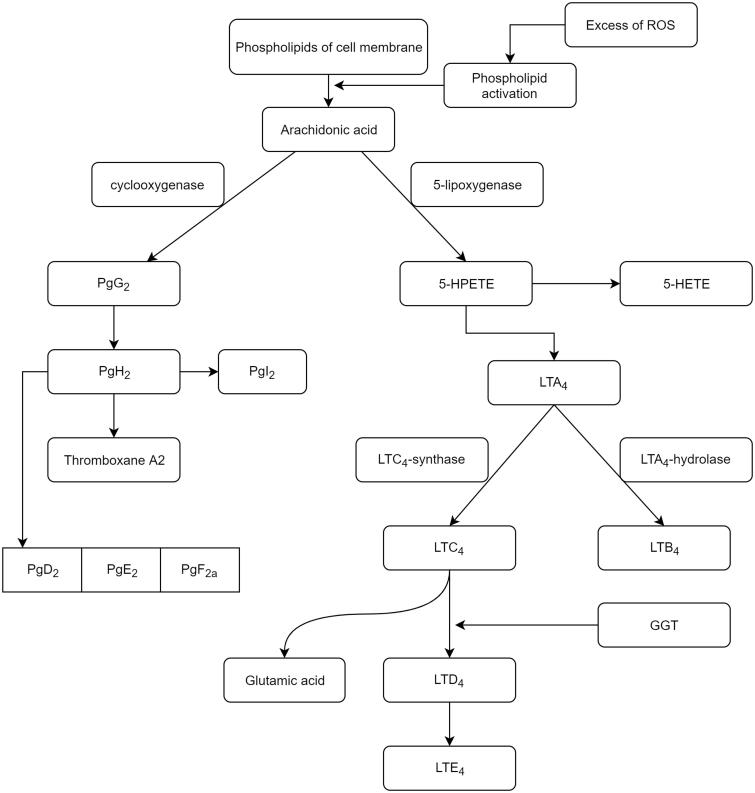
The metabolism of membrane arachidonic acid under oxidative stress conditions^11^. (5-HPETE: 5-hydroperoxyeicosatetraenoic; 5-HETE: 5-hydroxyeicosatetraenoic acid; LT: leukotriene; Pg: prostaglandin; GGT: gamma-glutamyltransferase).

The microbial presence at the diverticular level through chemostasis determines the migration of cells belonging to the phagocytic mononuclear blood system^5^. Neutrophils migrating into inflammatory outbreaks release phosphatases from lysosomes, a facilitator process for accessing cyclooxygenases and lipoxygenases to membrane polyunsaturated fatty acids, mainly arachidonic acid. If the activated pathway of cyclooxygenase leads ultimately to the formation of prostaglandins and thromboxane A2, then the pathway of activating lipoxygenases (5-, 12-, and 15-) synthesizes leukotrienes (Figure 2).

**Figure 3.**
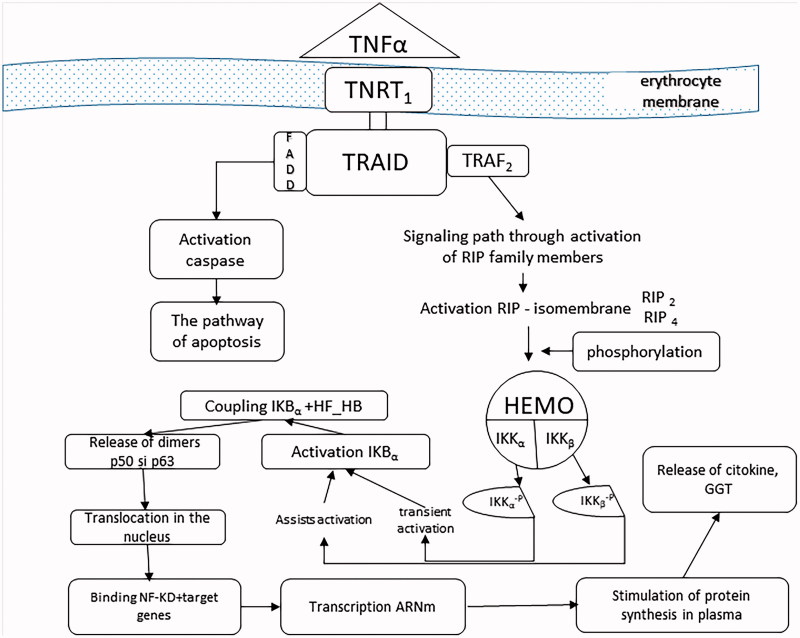
The process of activating the enzymatic synthesis of GGT, the source of its increasing circulating concentration.

In the leukotriene production process, GGT occurs in the conversion of LTC4 into LTD4^15^. Stimulation of expression of this enzyme is the consequence of activation of its synthesis, induced by TNF-α, also called cachectin, a product of cellular macrophages. TNF-α, a member of the superfamily of the same name, has the ability to regulate cell viability, neutrophil activation, IL1, and PgE2 synthesis^14^^,^^16^. In physiological concentrations, it has little effect on cultured human cells, but becomes toxic, directly under the conditions of excessive secretion. High concentrations of TNFα are produced by macrophage contact with bacterial products, IL_1_, and membrane lipopolysaccharides^17^. By binding TNFα to one of the two specific receptors on the TNFR_1_ and TNFR_2_ enterocyte membrane, their spatial disposition is modified, leading to the cascade activation of transcription of various proteins involved in cell proliferation, survival/apoptosis, or in the inflammatory response. In most cells, therefore, the ubiquitar TNRF_1_, the receptor through which it develops most of the biological functions induced, is distributed to the enterocytic membrane level. Following TNF_α_–TNFR_1_ coupling occurs the receptor trimerization, which allows it to couple to the DD domain of the TRADD activating protein (TNF) receptor protein. Hence, two signalling pathways are activated: the pathway of apoptosis involving TRADD coupling with FADD (Fas-associated protein with death domain) and the signalling pathway involving TRADD activation of TRAF_2_ (TNF receptor associated factor) (Figure 3).

In the intra-enterocyte protein activation process, an important role is played by the NF-κB cytoplasmic protein, discovered in 1980, as a pro-inflammatory factor, which under physiological conditions is inactive due to the presence of an inhibitor (IkB). Subsequent research has shown that NF-kB factor is a protein superfamily, with five members (p50 and p52 – the NH2-terminal fragments of the longer NF-κB1/p105 and NF-κB2/p100 proteins, respectively – p65 (RelA) c-Rel, and RelB), which act as a central regulator of immune, inflammatory and cell survival^2^^,^^18^. Numerous studies suggest that ROS may activate and/or suppress NF-κB depending on cell type and existing conditions^19^. Of the four modes by which the NF-kB factor can be activated, classical activation, canonical involves a sequence of two reactions which unfold of the cytoplasm.

a. To obtain an enterocytic inflammatory response, initially, some protein molecules involved in other functions and signalling pathways are stimulated, as are some of the seven members of the RIP family (receptor-interacting proteinkinase). They induce kN kinase inhibitor activation (IKK), which is composed of two catalytic subunits (IKK_α_/IKK_1_, IKK_β_/IKK_2_) and an essential modulator (NEMO/IKK_γ_). The catalytic subunits have in the COOH-terminal an identical hexapeptide sequence, called the NEMO binding domain (NDD), which allows them to assemble in NEMO. Dissociation of NEMO from NBD blocks the proinflammatory effect of FN-kB^18^. IKK can also be activated by oxidizing agents or by IL_1_. The activation consists of a phosphorylation reaction of the IKK catalytic subunits as a result of the adhesion of the initial modulator (NEMO) to the RIP proteins. Phosphorylation or very likely autophosphorylation of the catalytic subunits of IKK is induced by the oligomeric form of NEMO. The phosphorylated IKK_α_ catalytic subunit becomes capable of inducing FN-κB transient activation, while phosphorylation of the other IKK_β_ catalytic subunit confers sustainability of activation through the α isomembrane^19^^,^^20^ (Figure 3).

Subsequently, through the IKK_α_ phosphorylated component results in phosphorylation of the IkB inhibitor, which becomes active (IkBα), induces the degradation of the S26 proteosome, releasing the p50–p65 heterodimers and the p50 homodimer from the cytoplasmic NF-κB-IκB complex into the cytoplasm^10^^,^^18^^,^^20^. Released, NF-kB translocates into the nucleus of the enterocyte, binding to the target genes, amplifying the transcription and stimulating the synthesis of mRNA and by it of various types of proteins, including IL_1_, PgE_2_, collagen, and G-GTP-ase^21^^,^^22^. This assumes that the exposure of enterocytes to oxidative stress, but also to IL1, has an adaptive response to the increase in γ-GT expression^16^. The diversity of cellular responses induced by FN-kB activation are mainly dependent on the duration of activation of this transcription factor. Thus, prolonged stimulation can lead not to cell survival, but to its apoptosis^13^^,^^19^^,^^23^.

## Conclusions

Ubiquity of the GGT cellular distribution and its ability to significantly modulate redox sensitive cellular functions provide scientific support of its involvement in both inflammatory and proliferative cell processes but also as an oxidative stress marker.

The location of the predominant enzyme in brush border membranes explains the possibility of circulating levels in organ disorders that have such cells in their structure. For this reason, serum dosing results of GGT should be analysed in the clinical and paraclinical context and not as absolutised as having exclusively hepatocyte origin.
